# Inadequate Bioavailability of Intramuscular Epinephrine in a Neonatal Asphyxia Model

**DOI:** 10.3389/fped.2022.828130

**Published:** 2022-02-21

**Authors:** Sara K. Berkelhamer, Payam Vali, Jayasree Nair, Sylvia Gugino, Justin Helman, Carmon Koenigsknecht, Lori Nielsen, Satyan Lakshminrusimha

**Affiliations:** ^1^Department of Pediatrics, Seattle Children's Hospital, University of Washington, Seattle, WA, United States; ^2^Department of Pediatrics, University California Davis School of Medicine, Sacramento, CA, United States; ^3^Department of Pediatrics, University at Buffalo SUNY, Buffalo, NY, United States

**Keywords:** resuscitation, epinephrine, intramuscular, asphyxia, neonatal, low-resource

## Abstract

**Background:**

Over half a million newborn deaths are attributed to intrapartum related events annually, the majority of which occur in low resource settings. While progress has been made in reducing the burden of asphyxia, novel approaches may need to be considered to further decrease rates of newborn mortality. Administration of intravenous, intraosseous or endotracheal epinephrine is recommended by the Newborn Resuscitation Program (NRP) with sustained bradycardia at birth. However, delivery by these routes requires both advanced skills and specialized equipment. Intramuscular (IM) epinephrine may represent a simple, low cost and highly accessible alternative for consideration in the care of infants compromised at birth. At present, the bioavailability of IM epinephrine in asphyxia remains unclear.

**Methods:**

Four term fetal lambs were delivered by cesarean section and asphyxiated by umbilical cord occlusion with resuscitation after 5 min of asystole. IM epinephrine (0.1 mg/kg) was administered intradeltoid after 1 min of positive pressure ventilation with 30 s of chest compressions. Serial blood samples were obtained for determination of plasma epinephrine concentrations by ELISA.

**Results:**

Epinephrine concentrations failed to increase following administration *via* IM injection. Delayed absorption was observed after return of spontaneous circulation (ROSC) in half of the studies.

**Conclusions:**

Inadequate absorption of epinephrine occurs with IM administration during asphyxial cardiac arrest, implying this route would be ineffective in infants who are severely compromised at birth. Late absorption following ROSC raises concerns for risks of side effects. However, the bioavailability and efficacy of intramuscular epinephrine in less profound asphyxia may warrant further evaluation.

## Introduction

Despite advances in the delivery of both maternal and newborn care, an estimated 2.4 million infants do not survive their first month of life (1). Nearly $\frac{1}{4}$ of neonatal deaths can be attributed to intrapartum events or asphyxia, resulting in an estimated 0.58 million deaths annually (2). Beyond mortality are concerns for long term morbidity associated with intrapartum events. The World Health Organization estimates that between 4 and 9 million newborns are impacted by asphyxia annually with risks of long term developmental delays associated with physiologic compromise at birth (3, 4). These data may even underestimate the incidence with the challenges of identifying cases in under resourced areas (5, 6). The burden of disease falls greatest on low and middle income countries where the majority of early newborn deaths occur (5).

While reduction in morbidity and mortality associated with birth asphyxia requires improvement in all levels of perinatal care, improved resuscitation and stabilization of asphyxiated infants could impact millions of infants worldwide. Resuscitation efforts at birth play a critical role in determining outcomes, influencing both risk of mortality and incidence of neurodevelopmental disability (4, 7, 8). Access to training and materials to provide simple resuscitative care (through programs including *Helping Babies Breathe*) has been shown to improve outcomes in low resource settings with reduction of early mortality and stillbirth rates (9). Despite these advances, outcomes following intrapartum related events remain a concern. Notably, the annual rate of reduction for neonatal mortality lags significantly behind rates of reduction for maternal and under 5 child mortality (10, 11). Additional simple measures to improve the care of infants compromised at birth may be needed to further reduce the burden.

The Newborn Resuscitation Program (NRP) utilizes recommendations provided by the International Liaison Committee on Resuscitation (ILCOR) to define an algorithm for care of compromised newborns. This program is currently used in over 130 countries worldwide and influences the care of millions of infants at delivery. These guidelines recommend administration of epinephrine by umbilical venous catheter (UVC), intraosseous (IO) needle, or endotracheal tube (ETT) with sustained bradycardia despite effective ventilation and chest compressions (12). However, these routes of administration require advanced skills, specialized equipment and take several minutes to secure even in well-resourced settings where training and equipment are readily available (13, 14). Simplified algorithms, including *Helping Babies Breathe*, provide guidance for basic resuscitative care. However, intramuscular (IM) epinephrine could be administered with limited training, use of commonly accessible resources and with limited interruption of resuscitative efforts. As every minute delay potentially impacts outcomes with asphyxia (15), a simple, efficient mode of epinephrine delivery has potential application in the context of both HBB or NRP when access cannot be obtained.

While epinephrine has inotropic, lusitropic, and chronotropic actions, its vasoconstrictor properties mediated by α-adrenergic receptors are primarily responsible for its effectiveness in CPR (16). Administration of epinephrine is believed to induce intense peripheral vasoconstriction resulting in elevated systemic vascular resistance and an increase in coronary perfusion pressure to improve coronary flow (17). IM epinephrine has known clinical efficacy and is widely used in the treatment of anaphylaxis, suggesting this route of delivery might also be considered with resuscitation. The ease of administration makes this approach a practical and efficient option for providers at all levels, an important consideration in less resourced environments where attendants skilled in intubation or line placement may not be available. While studies in a pediatric swine model suggest IM epinephrine may promote return of spontaneous circulation (ROSC) after cardiac arrest (18), the absorption and pharmacokinetics associated with this route of administration in the context of asphyxia remain unknown. Using a well-established ovine model of neonatal asphyxial arrest, we sought to determine bioavailability and pharmacokinetics associated with administration of IM epinephrine.

## Materials and Methods

### Animal Studies

Four term gestation lambs (141–142 days) were used for this case series. Our protocol was performed on extra, unassigned lambs with a specific interest in comparing an alternative route of epinephrine administration to our prior studies evaluating bioavailability and pharmacokinetics with delivery *via* IO needle, UVC, or ETT (19, 20). ARRIVE guidelines were followed as possible, however our cohort was limited to 4 lambs and was not randomized or registered as a preclinical trial.

All animal work was approved by the Institutional Animal Care and Use Committee at the State University of New York at Buffalo (Protocol #PED10085N, approved 5.10.2018). Time dated term (140**–**141 day gestation) pregnant ewes were obtained from Newlife Pastures (Varysburg, New York, USA). Following an overnight fast, the ewes were anesthetized with intravenous diazepam and ketamine, intubated with a 10.0-mm cuffed ETT and ventilated with 21% oxygen and 2–3% isoflurane at 16 breaths/min. Ewes were continuously monitored with pulse oximetry and an end tidal CO_2_ monitor. Cesarean section was performed on the anesthetized ewe with partial exteriorization of fetal lambs. Lambs were intubated and excess fetal lung fluid in the ETT was drained by gravity to simulate loss of lung liquid with labor. Thereafter, the ETT was occluded to prevent gas exchange during gasping in the asphyxia period. Fetal lambs were instrumented as described previously (21–23), with heparinized catheters placed into the right carotid artery and jugular vein for blood pressure measurements, blood draws to measure plasma epinephrine concentrations and preductal arterial blood gases. A 2-mm flow probe (Transonic Systems Inc., Ithaca, NY) was placed around the left carotid artery. A left thoracotomy was performed and a 4-mm flow probe was placed around the left pulmonary artery. The thoracotomy was closed in layers. ECG leads were attached at the right axilla, left axilla, and right inguinal area (3-lead EKG). The ECG100C (Biopac Systems, Inc.) was used with Acknowledge Software to record tracings of leads I, II, and III. Preductal arterial oxyhemoglobin saturation was monitored with a pulse oximeter placed on the right forelimb of the lamb (Masimo, Irvine, CA). Following instrumentation, a “baseline” blood gas and sample for epinephrine concentration was obtained. The umbilical cord was subsequently occluded and cut, and the lambs were moved from the maternal abdomen to the radiant warmer. During the asphyxia period, an umbilical arterial catheter was inserted to measure continuous invasive blood pressures. A low umbilical venous catheter was inserted 2 cm below the skin (and secured after confirming blood drawing back into the catheter).

### Experimental Protocol

A 5-min period of asystole was observed prior to initiating resuscitation. Asystole was defined by the absence of carotid blood flow, arterial blood pressure, and heart rate (by auscultation). Resuscitation began by removing the ETT occluder and providing positive pressure ventilation (PPV) with 21% oxygen by means of a T-piece resuscitator at pressures of 35/5 cm H_2_O (to delivery standard volumes and ventilation) at a rate of 40 breaths/min (24). Following 30s of ventilation, chest compressions at a compression-to-ventilation ratio of 3:1 were commenced with a simultaneous increase in inspired oxygen to 100%. Intramuscular epinephrine was administered into the deltoid muscle at 1 min from onset of resuscitation. A high dose of 0.1 mg/kg (1:1,000 concentration, 1 mg/mL) was utilized as we assumed there would be challenges with absorption and wanted to parallel prior published studies evaluating this route in an arrest model (18). Only a single dose was administered to simplify evaluation of pharmacokinetics. Synchronized chest compressions and PPV were continued until ROSC was achieved, defined as a sustained, perfusing rhythm with heart rate above 100 beats per minute.

**Figure 1:**
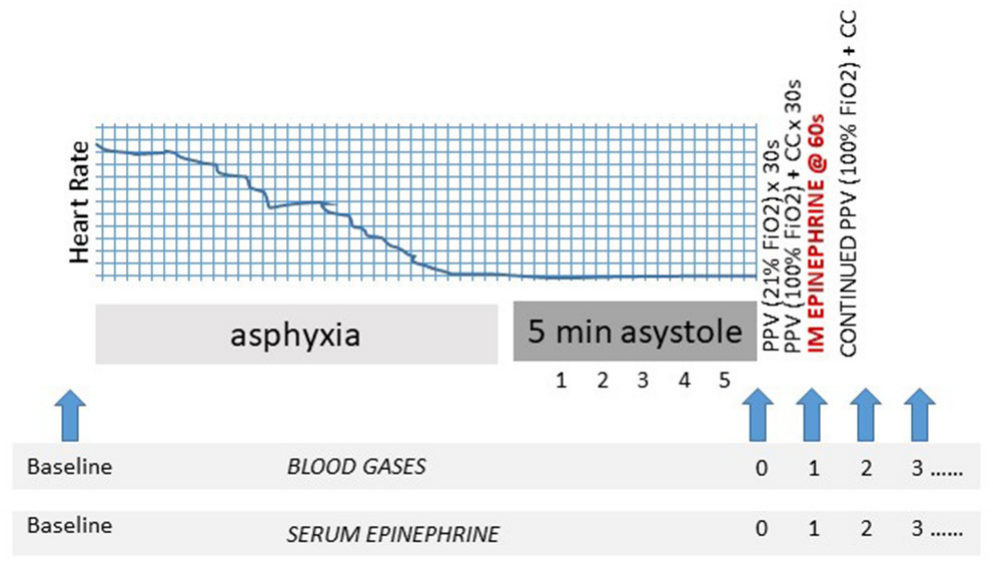
Experimental design. Schematic identifying resuscitation protocol and blood sampling (blue arrows). CC, to chest compression; FiO_2_, fraction of inspired oxygen; PPV, positive pressure ventilation; s, second.

An “arrest” arterial blood sample was obtained following 5 min of asystole but prior to initiation of resuscitation (time 0 of resuscitation). Thereafter, serial blood sampling was performed at minute 1–5, 7, 9, and 15 for blood gas analysis and epinephrine levels. The 1-min sample was drawn immediately prior to injection of epinephrine (Figure 1). Arterial blood samples were analyzed using a radiometer blood gas analyzer (ABL 800 FLEX, Denmark). Plasma epinephrine concentrations were analyzed by ELISA (Eagle Biosciences, Nashua, NY) with a limit of detection as defined by the assay of < 0.005 ng/mL.

### Statistical Analysis

Continuous variables were analyzed by ANOVA with Tukey's *post-hoc* analysis. GraphPad Prism (San Diego, CA) was used for statistical analysis with significance defined as *p* < 0.05.

## Results

### Demographics and Asphyxia

Four lambs were studied, including one singleton female, one singleton male, one twin female and one twin male with an average weight of 3.6 ± 0.9 kg. Baseline blood gases were comparable to those of prior experimental lambs with the blood gas at arrest demonstrating severe metabolic acidosis, hypercapnea, and elevated lactate levels (Table 1) (22). Time to asystole was a median (range) of 12.6 (8–15.5) min, also consistent with prior publications in the model (22).

**Table 1:** Blood gases at baseline and arrest.

		**Blood gas at baseline**			**Blood gas at arrest**		
***Route***	***N***	**pH**	**CO2**	**Lactate**	**pH**	**CO2**	**Lactate**
*IM*	4	7.19 ± 0.08	66 ± 12	3.3 ± 0.6	6.81 ± 0.08	126 ± 30	12.5 ± 4.4
UVC*	11	7.10 ± 0.09	67 ± 7	4.9 ± 3.6	6.80 ± 0.09	146 ± 25	14.6 ± 6.7
ETT*	11	7.16 ± 0.14	68 ± 7	4.8 ± 3.5	6.85 ± 0.04	132 ± 17	14.8 ± 5.1

*Baseline samples were obtained following instrumentation but prior to asphyxia. Arrest samples were obtained after 5 min of asphyxia*.

* *Experimental data from Vali et al. (22). N represents the number of lambs, Data are mean ± SD. IM, intramuscular; UVC, umbilical venous catheter; ETT, endotracheal tube*.

### Plasma Epinephrine Concentrations

**Figure 2:**
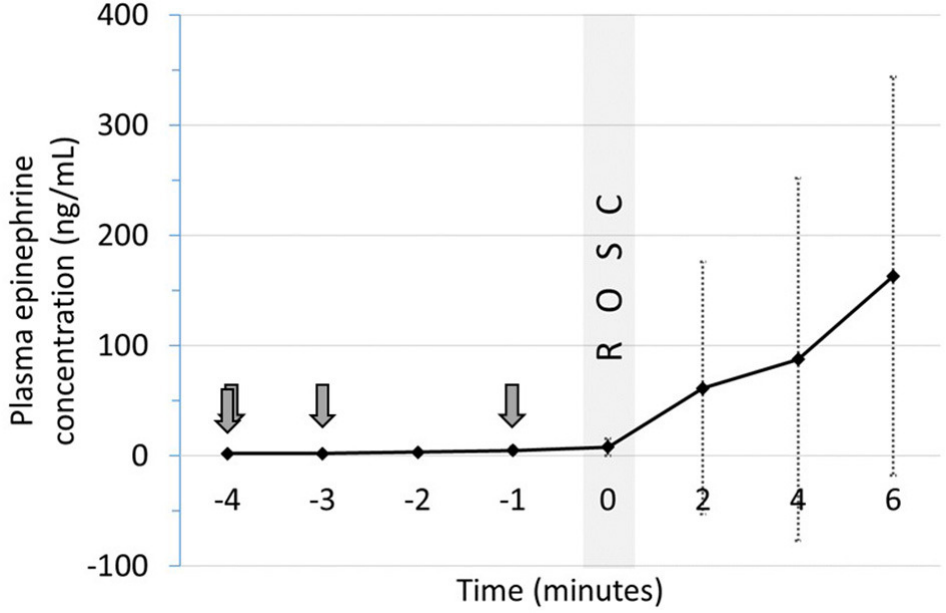
Plasma epinephrine concentrations following IM epinephrine in relation ROSC. Arrows indicate time of epinephrine administration. Data are mean ± SEM. ROSC, return of spontaneous circulation.

**Figure 3:**
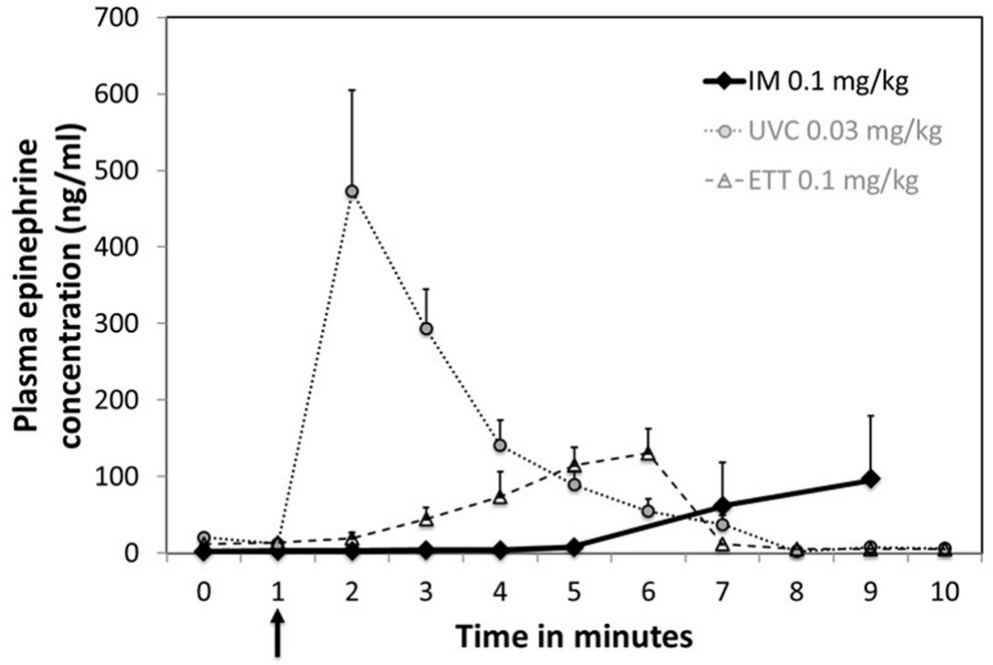
Plasma concentrations following a single dose of IM epinephrine. Arrow indicates time of epinephrine administration. Data are mean ± SEM. Mean concentration for IM at 15 min was 216.2 ± 91.1 ng/mL. UVC and ETT concentration as previously published by Vali et al. (19) are included for comparison.

Plasma epinephrine concentrations were evaluated at baseline (prior to asphyxia), arrest (0 min) and serially during resuscitation. There was no significant increase in plasma concentrations prior to ROSC with IM epinephrine administration (Figure 2). Increased concentrations were observed in 2 animals several minutes after ROSC (Supplementary Table 1). While epinephrine concentrations did not increase significantly following IM administration (Figure 3), delayed absorption occurred.

### Return of Spontaneous Circulation (ROSC)

All 4 animals achieved ROSC with IM administration with time to ROSC at a median (range) of 3.95 (1.7–4.7) min. Mean carotid and pulmonary artery flows, systolic and diastolic blood pressures at and 2 min following ROSC are presented in Table 2.

**Table 2:** Hemodynamics at and following ROSC.

	**ROSC**	**ROSC + 2 min**
CA flow (ml/min)	11 ± 6	27 ± 13
PA flow (ml/min)	6 ± 3	37 ± 18
SBP (mm Hg)	56 ± 38	77 ± 25
DBP (mm Hg)	31 ± 22	55 ± 18

*Mean ± SD of carotid (CA) and pulmonary (PA) artery blood flows, and systolic (SBP) and diastolic (DBP) blood pressures at and 2 min following ROSC*.

## Discussion

Epinephrine is recommended in resuscitation algorithms utilized in well-resourced settings, with options for delivery *via* intravascular, intraosseous, and endotracheal routes. At present, the potential application of epinephrine in algorithms of care in less resourced environments, where the burden of asphyxia occurs, remains poorly defined. Complex routes of delivery limit broad use of this potentially life-saving medication as resuscitation in low resource settings (as taught in *Helping Babies Breathe* or the World Health Organization's *Essential Newborn Care*) would not include intubation or placement of umbilical or intraosseous access. In light of publications suggesting efficacy (18), we sought to investigate the concept of low cost, simplified delivery of epinephrine *via* intramuscular injection. However, our exploratory studies in a bovine asphyxia model evaluating pharmacokinetics with IM administration identified inadequate absorption of epinephrine until after ROSC. This result is consistent with hemodynamic redistribution that occurs with cardiac arrest and prioritization of perfusion to critical organs over peripheral tissues.

Despite epinephrine's controversial impact on long term outcomes (25, 26), early administration has been shown to influence timing of ROSC in both animals and clinical models supporting our interest in simplified and efficient delivery (22, 27, 28). Animal studies further identify that chest compressions alone are inadequate in improving cerebral blood flow and that administration of epinephrine improves both coronary and cerebral perfusion with higher probability of ROSC (29–31). However, compromised infants can only benefit from administration of epinephrine if IV, IO or ET access can be obtained.

This scenario can occur in both resourced and low resourced settings. As example, a letter to the editor of *Resuscitation* described the use of IM epinephrine in an infant with perinatal asphyxia in Italy as access was not able to be obtained at the referral hospital (32). The infant received only IM epinephrine during resuscitation and survived without morbidity after severe perinatal acidosis. While it is unclear whether this dose of epinephrine influenced outcomes for this patient, this publication highlights the possibility and confirms that use of IM epinephrine has been considered in the stabilization of compromised newborns. We are unaware, however, of clinical settings where this is a common practice.

Additional data on the role of IM epinephrine in cardiopulmonary resuscitation exists from studies performed by Mauch et al. in a pediatric swine model of ropivacain-induced cardiac arrest (18). The authors report comparable rates of survival in pigs administered epinephrine by an IM route (0.1 mg/kg) as compared to IV dosing (0.01 mg/kg). In addition, they observed higher rates of survival and earlier return of spontaneous circulation (ROSC) with IM epinephrine as compared to normal saline controls. However, epinephrine concentrations were not evaluated in these studies. As our study failed to demonstrate absorption of epinephrine prior to ROSC with IM administration in asphyxia, the reported efficacy of this route in the piglet studies may be due to differences in the two models. Cardiopulmonary resuscitation (CPR) was initiated with the onset of circulatory arrest in the piglet model, with arrest defined as mean arterial pressures (MAP) of <25% of initial value, representing a clinically pulseless state. The piglets were unlikely to be as profoundly compromised, as our model utilizes a 5-min period of asystole. However, blood gases were not provided limiting our ability to compare the degree of asphyxia.

We speculate that the peripheral vasoconstriction associated with our model was less pronounced in the piglet study, facilitating perfusion and absorption of IM epinephrine during resuscitation. While our results imply IM epinephrine would be ineffective and risk delayed absorption and side effects, its role in the context of less profound asphyxia (including bradycardia without arrest) may still be worthy of evaluation.

Limitations of our model include the translation of findings in a lamb to human, with recognition of the potential contributions of hypovolemia and compromised cardiac glycogen stores in clinical settings (33, 34). This brief research study was also limited in it non-randomized design as studies were performed on extra, unassigned lambs as available. This approach has potential for selection bias as the experimental treatment was predetermined. In addition, only 4 studies were performed in this small, exploratory series. However, the results of our studies prior to ROSC were highly consistent, making it difficult to rationalize use of additional animals as added studies were not expected to influence our assessment of bioavailability. Similarly, this small exploratory study did not evaluate efficacy, impact on hemodynamics or ROSC, as these were all outcomes that did not seem appropriate to assess in absence of documented absorption. Finally, beyond concerns for late absorption, the safety of IM administration was not evaluated in our model. IM administration in the context of inadequate muscle perfusion risks local tissue injury and necrosis.

We would have anticipated delayed circulatory recovery, however the four animals achieved ROSC at a time comparable to that observed with ETT dosing, implying that effective CPR, rather than increased concentration of epinephrine, may have played a greater role in determining outcomes. Indeed, published studies in the ovine asphyxia model found that 6/13 animals achieved ROSC *prior* to administration of epinephrine when it was delayed to 6 min (35) and that epinephrine concentrations in absence of exogenous delivery was comparable to that observed with IM dosing (5–20 ng/mL) (22). It remains plausible that historical studies in UVC and ETT may have achieved ROSC in absence of epinephrine had it not been administered early (22). Indeed, hemodynamic data during chest compressions in the asphyxia model failed to demonstrate increase in systolic or diastolic blood pressure, or carotid blood flow following epinephrine administration with speculation that adenosine triphosphate depletion may contribute to the lack of effect (36, 37).

## Conclusion

Studies in the neonatal ovine model suggest that inadequate absorption of epinephrine occurs with intramuscular administration during asphyxia. While a simplified route of delivery might be of interest, the lack of increase in epinephrine concentrations until several minutes after ROSC both questions the relevance of this route of administration as well as its safety. However, the bioavailability and efficacy of IM epinephrine in the context of less profound asphyxia may still warrant investigation.

## Data Availability Statement

The raw data supporting the conclusions of this article will be made available by the authors, without undue reservation.

## Ethics Statement

The animal study was reviewed and approved by Institutional Animal Care and Use Committee at the State University of New York at Buffalo.

## Author Contributions

SB and SL: conceptualization. SB, SL, SG, and CK: methodology. SB, SL, and CK: formal analysis. SB, JN, SG, JH, CK, LN, PV, and SL: investigation and resources SB, PV, JN, and CK: data curation. SB: writing—original draft preparation. SB, JN, and SL: writing—review and editing. SG and CK: project administration. All authors contributed to the article and approved the submitted version.

## Funding

This research was funded by the NIH HD 072929 (SL) and American Academy of Pediatrics Neonatal Resuscitation Program (SL and SB).

## Conflict of Interest

The authors declare that the research was conducted in the absence of any commercial or financial relationships that could be construed as a potential conflict of interest. The reviewer P-YC declared a past collaboration with one of the authors SL to the handling editor BN.

## Publisher's Note

All claims expressed in this article are solely those of the authors and do not necessarily represent those of their affiliated organizations, or those of the publisher, the editors and the reviewers. Any product that may be evaluated in this article, or claim that may be made by its manufacturer, is not guaranteed or endorsed by the publisher.
