# Supplementary material for: Inadequate Bioavailability of Intramuscular Epinephrine in a Neonatal Asphyxia Model
Source: Front Pediatr. 2022 Feb 21;10:828130. doi: 10.3389/fped.2022.828130 (PMC8899212; doi:10.3389/fped.2022.828130)
Supplement: Supplementary file 1 [file Table_1.DOCX]

**Supplemental Table 1. Serial plasma epinephrine concentrations (ng/mL).**

|  |  | **0** | 1  EPI | **2** | **3** | **4** | **5** | **7** | **9** | **15** |
| --- | --- | --- | --- | --- | --- | --- | --- | --- | --- | --- |
| ***Exp. # 1*** | 0.01 | 3.6 | 6.8 | ***4.7*** | ***5.3*** | ***2.4*** | ***1.8*** | ***3.1*** | ***3.3*** | ***6.5*** |
| ***Exp. # 2*** | ND | 3.0 | 3.6 | 3.3 | 3.5 | ***6.1*** | ***7.0*** | ***7.5*** | ***6.8*** | ***2.9*** |
| ***Exp. # 3*** | 0.5 | 1.1 | 1.3 | 1.1 | 1.5 | 1.2 | ***1.7*** | ***3.9*** | ***3.7*** | ***334*** |
| ***Exp. # 4*** | 0.2 | 0.8 | 1.6 | 3.4 | 5.1 | 8.1 | ***18.7*** | ***233*** | ***334*** | ***304*** |
| ***AVG***  ***(SEM)*** | 0.2  (0.1) | 2.1  (0.7) | 3.3  (1.3) | 3.1  (0.8) | 3.8  (0.9) | 4.4  (1.6) | 7.3  (4.0) | 61.9  (57.1) | 87.1  (82.5) | 216.2  (91.1) |

Epinephrine administered at 1 minute of resuscitation after 30 seconds of positive pressure ventilation (PPV) followed by PPV with chest compressions x 30 seconds. EPI epinephrine; exp experiment; ND not detectable; AVG average; SEM error of the mean. ***Italicized*** values represent concentrations after ROSC.
